# Association of hemoglobin levels with radiographic progression in patients with rheumatoid arthritis: an analysis from the BRASS registry

**DOI:** 10.1186/s13075-023-03068-w

**Published:** 2023-05-27

**Authors:** Nancy Shadick, Owen Hagino, Amy Praestgaard, Stefano Fiore, Michael Weinblatt, Gerd Burmester

**Affiliations:** 1 Division of Rheumatology, Inflammation, and Immunity, Brigham and Women’s Hospital, Boston, MA USA; 2 Sanofi, Research and Development, Bridgewater, NJ USA; 3 Global Medical Affairs, Biostatistics, Cambridge, MA USA; 4 Department of Rheumatology and Clinical Immunology, Charité-Universitätsmedizin, Berlin, Germany

**Keywords:** Rheumatoid arthritis, Biological therapy, Methotrexate, Tumor necrosis factor inhibitors

## Abstract

**Background:**

To evaluate baseline hemoglobin (Hb) and radiographic progression over time in patients enrolled in the Brigham and Women’s Rheumatoid Arthritis Sequential Study (BRASS) registry.

**Methods:**

The BRASS is a prospective observational registry of patients with rheumatoid arthritis. BRASS Hb data and total sharp score data were matched with the main BRASS patients. Hb at baseline was categorized per the World Health Organization guidelines. Mean Hb, mean total sharp score, and mean changes over time from baseline to month 120 were summarized (overall, by low/normal Hb, and by current medication at baseline). All analyses were descriptive.

**Results:**

Out of the total (*N* = 1114) rheumatoid arthritis patients included in the analysis, patients with low Hb at baseline (*n* = 224 [20%]) had longer disease duration and higher disease activity and reported more pain compared with patients with normal Hb at baseline (*n* = 890 [80%]). Patients with low Hb at baseline continued to have lower Hb than patients with normal Hb throughout 10 years; although, on average, patients in the low Hb subgroup exhibited a steady increase in Hb levels. A larger increase in total sharp score over time was observed for patients with low Hb than for patients with normal Hb. No meaningful differences potentially attributable to medication at baseline were detected.

**Conclusions:**

Patients with low Hb levels at baseline tended to have increased radiographic progression as measured by total sharp score compared with patients with rheumatoid arthritis having normal Hb levels. Patients with low Hb experienced sustained improvements in Hb levels over time, regardless of the class of medication used.

**Trial registration:**

ClinicalTrials.gov NCT01793103.

**Supplementary Information:**

The online version contains supplementary material available at 10.1186/s13075-023-03068-w.

## Background

Rheumatoid arthritis (RA) is an autoimmune and chronic inflammatory disease that affects the joints and extra-articular organs [1]. Traditionally, RA has been monitored with clinical and radiographic data along with clinical (joint) examination. Although it would be useful to quantitatively assess the disease activity or risk of joint damage in routine clinical practice, very few rheumatologists in the USA use composite disease activity measures as those are time-consuming [2]. Moreover, routinely used assessments can be entirely subjective and non-specific (e.g., laboratory tests providing “false negative” results) and may have little prognostic value (e.g., radiographs) [3]. Therefore, identification of an alternative tool is needed to shed light on disease activity and progression.

Anemia affects about 15% to 47% of patients with RA [4, 5, 6]. A study on the Swiss Clinical Quality Management database reported that RA patients with anemia had more severe radiological progression, whereas this association between anemia and erosion progression was independent of established disease activity scores [5]. Furthermore, RA patients with anemia demonstrated a higher disease activity, structural damage, and reduced joint function compared with non-anemia patients [4]. Low hemoglobin (Hb) levels were found to be an early indicator of active clinical or subclinical inflammatory disease, possibly associated with RA disease severity [7, 8] and DAS28-C-reactive protein (CRP) was found to be an independent predictor of radiographic joint damage progression [9]. Considering these evidences, evaluation of Hb levels may help in identifying patients with rapid radiological progression.

Therefore, the present study evaluated baseline Hb as a prognostic test for the radiographic progression over time in the RA patient population enrolled in the Brigham and Women’s Rheumatoid Arthritis Sequential Study (BRASS) registry.

## Methods

### Study design

The BRASS (ClinicalTrials.gov identifier, NCT01793103) is a single-center, prospective, observational, and longitudinal real-world registry. It includes clinical and patient-reported data of > 1500 adult patients with RA registered at the Robert Breck Brigham Arthritis Clinic in Boston, MA, USA [10]. The study began enrolment in March 2003, and the retention rate was estimated to be approximately 80% to 90%. The current analysis was performed based on the first and the last visit dates (from 2003 to 2021) to evaluate patients’ overall data enrolled in the BRASS registry. Aim of the analysis was to determine baseline Hb as a prognostic test for patients in the BRASS registry by evaluating Hb and total Sharp score (TSS) over time (by Hb subgroups and current medication at baseline).

### Patients

Patients were considered eligible to enroll in the registry if they were 18 to 70 years old with an RA diagnosis made by a board-certified rheumatologist and were registered at the Robert Breck Brigham Arthritis Center. Patient confidentiality was maintained by assigning each patient a clinical subject identification number upon enrollment in the BRASS registry. The present study population represents a subset of the BRASS cohort where the BRASS Hb and TSS data fields were matched to the core BRASS data according to the patient ID and a visit date within 15 days of the Hb and TSS collection date.

### Measures and data collection

Hemoglobin at baseline measurements was stratified as low (Hb < 120 g/L for women or < 130 g/L for men) and normal (Hb ≥ 120 g/L for women or ≥ 130 g/L for men) based on the World Health Organization (WHO) guidelines. Upon enrollment, patient demographic and clinical information, functional status, disease activity, comorbidity, laboratory testing, and hand radiographs were obtained at baseline. A physical examination, including joint examination and assessment of pain and disease activity, was retrieved from the BRASS registry.

### Statistical analysis

Descriptive analyses were used to summarize the demographic and baseline characteristics of patients, stratified by low and normal Hb. Continuous variables are reported as mean and standard deviation (SD). The statistical significance (*P*-value) of the difference between the low and normal Hb groups was obtained using *t*-tests for equality of variance and Satterthwaite’s estimation for unequal variances. Categorical variables are reported as number (*n*) and percentage (%). The statistical significance (*P*-value) of the difference between the Hb groups was obtained using chi-square tests or Fisher’s exact tests. The mean Hb and the mean TSS from baseline to month 120 by Hb subgroups (low/normal) and by current medication (anti-tumor necrosis factor [TNF], biologic disease-modifying anti-rheumatic drug (DMARD), and non-biologic DMARDs) at baseline were analyzed. Likewise, mean changes in Hb and TSS through 120 months were analyzed for overall patients and subgroups (Hb at baseline subgroups [low/normal] and current medication) at baseline. SAS v 9.4 was used for all analyses.

The study protocol and informed consent documents were reviewed and approved by the institutional review board of Brigham and Women’s Hospital (approval number: 2002P001762).

## Results

### Demographics and baseline disease characteristics

In total, 1114 patients with RA were included in the analysis. Patients were stratified to low (*n* = 224; 20.11%) and normal (*n* = 890; 79.89%) subgroups based on Hb levels at baseline.

Patients with low Hb at baseline were likely to be older (mean [SD]: 60.38 [14.61] vs 55.53 [13.74] years old; *p* < 0.0001), less likely to be Caucasians (187/219 [85.39%] vs 838/889 [94.26%]; *p* < 0.0001), had a longer disease duration (14.05 [12.83] vs 11.71 [11.41] years; *p* = 0.0134), a higher mean (SD) DAS28-CRP4S (4.46 [1.65] vs 3.53 [1.57]; *p* < 0.0001), and reported more number of swollen (8.83 [7.85] vs 5.70 [6.89]; *p* < 0.0001) and painful joints (9.71 [8.74] vs 6.52 [7.36]; *p* < 0.0001) than patients with normal Hb (Table 1).

**Table 1:** Baseline demographic and disease characteristics by Hb subgroup

Characteristic	Low Hb (*N* = 224)	Normal Hb (*N* = 890)	*P*-value
Age (years)^a^	*N* = *224*	*N* = *890*	< 0.0001
	60.38 (14.61)	55.53 (13.74)	
Male^b^, *n* (%)	*N* = 224	*N* = 890	0.3508
	36 (16.07)	167 (18.76)	
Caucasians^b^, *n* (%)	*N* = 219	*N* = 889	< 0.0001
	187 (85.39)	838 (94.26)	
Body mass index^a^, (kg/m^2^)	*N* = 208	*N* = 832	0.4822
	26.99 (6.34)	26.65 (5.58)	
Disease duration^a^, mean (SD) years	*N* = 223	*N* = 890	0.0134
	14.05 (12.83)	11.71 (11.42)	
Seropositive^b^, *n* (%)	*N* = 214	*N* = 832	0.6315
	150 (70.09)	569 (68.39)	
RF positive^b^, *n* (%)	*N* = 212	*N* = 808	0.5586
	126 (59.43)	498 (61.63)	
Anti-CCP positive^b^, *n* (%)	*N* = 212	*N* = 823	0.1578
	140 (66.04)	500 (60.75)	
DAS 28-CRP4S^a^, mean (SD)	*N* = 195	*N* = 791	< 0.0001
	4.46 (1.65)	3.53 (1.57)	
Total swollen joints^a^, mean (SD)	*N* = 224	*N* = 889	< 0.0001
	8.83 (7.85)	5.70 (6.89)	
Total painful joints^a^, mean (SD)	*N* = 224	*N* = 889	< 0.0001
	9.71 (8.74)	6.52 (7.36)	
Medicines ever taken, *n* (%)			
Methotrexate^b^	163 (72.77)	697 (78.31)	0.077
Anti-TNF^b^	93 (41.52)	406 (45.62)	0.27
Plaquenil^b^	135 (60.27)	531 (59.66)	0.8689
Biologic DMARD	97 (43.30)	412 (46.29)	0.4222
Non-biologic DMARD	210 (93.75)	848 (95.28)	0.3486
Non-biologic DMARD, not including MTX or Plaquenil^b^	121 (54.02)	414 (46.52)	0.0446
Steroid^b^	182 (81.25)	713 (80.11)	0.7018
Current medicines, *n* (%)			
Methotrexate^b^	107 (47.77)	491 (55.17)	0.0471
Anti-TNF^b^	67 (29.91)	315 (35.39)	0.1223
Anti-TNF and MTX^b^	32 (14.29)	170 (19.10)	0.0945
Plaquenil^b^	41 (18.30)	163 (18.31)	0.997
NSAIDs^b^	133 (59.38)	439 (49.33)	0.0072
Any DMARD^b^	192 (85.71)	804 (90.34)	0.0445
Biologic DMARD^b^	74 (33.04)	347 (38.99)	0.1005
Non-biologic DMARD^b^	159 (70.98)	673 (75.62)	0.1538
Non-biologic DMARD, not including MTX or Plaquenil^b^	47 (20.98)	139 (15.62)	0.0543
Steroid^b^	74 (33.04)	252 (28.31)	0.1651

Baseline hemoglobin < 120 g/L for women or < 130 g/L for men is considered as low Hb as per the World Health Organization guidelines

*N*, number of patients by baseline hemoglobin status; number: number of patients for each characteristic by baseline hemoglobin status. Percentages are based on “number”

*CCP* Cyclic citrullinated peptide, *DMARD* Disease-modifying antirheumatic drug, *Hb* Hemoglobin, *MTX* Methotrexate, *NSAID* Nonsteroidal anti-inflammatory drug, *RF* Rheumatoid factor, *SD* Standard deviation, *TNF* Tumor necrosis factor

^a^*P*-value is obtained using a *t*-test for equality of variance. In case equality of variance assumption is not met, Satterthwaite’s *P*-value is provided

^b^*P*-value is obtained using chi-square test. In case expected cell frequency is < 5, Fisher’s exact test is used

At baseline, patients with low Hb were less likely to take methotrexate (MTX) (163 [72.77%] vs 697 [78.31%]) and non-biologic DMARDs (210 [93.75%] vs 848 [95.28%]) compared with patients with normal Hb. A lower proportion of patients with low Hb were currently taking anti-TNF + MTX (32 [14.29%] vs 170 [19.10%]) and biologic DMARDs (74 [33.04%] vs 347 [38.99%]) than patients with normal Hb. However, a higher proportion of patients with low Hb were currently on nonsteroidal anti-inflammatory drug (NSAID) (133 [59.38%] vs 439 [49.33%]) and non-biologic DMARD (not including MTX or Plaquenil; 47 [20.98%] vs 139 [15.62%]) than normal Hb patients.

### Hemoglobin over time by Hb subgroups and current medication at baseline

#### Hemoglobin over time by hemoglobin subgroups

**Fig. 1:**
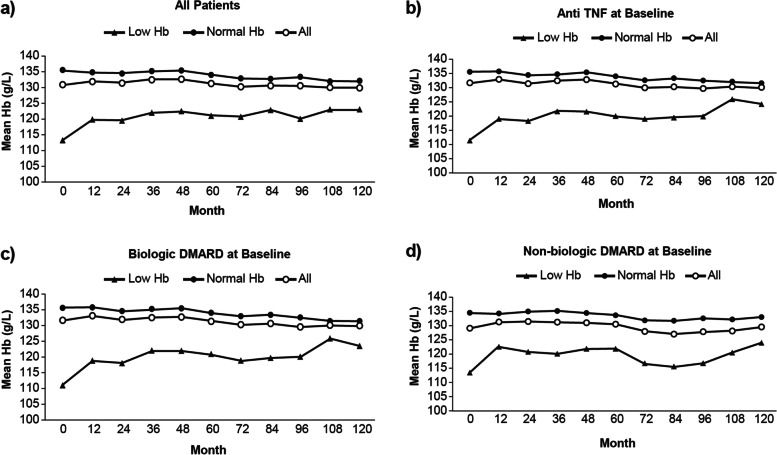
Mean Hb over time treated with various classes of medications by Hb subgroup DMARD, disease-modifying anti-rheumatic drug; Hb, hemoglobin; TNF, tumor necrosis factor

Overall, little change was observed in the mean Hb from baseline (*n* = 1114; 130.82 g/L) to 10 years (*n* = 186; 129.98 g/L) in patients with RA (Fig. 1). Patients with low Hb at baseline (*n* = 224; 113.12 g/L vs *n* = 890; 135.28 g/L) continued to have lower Hb than patients with normal Hb at baseline throughout 10 years (*n* = 42; 123.07 g/L vs *n* = 144; 132.00 g/L), respectively. On average, patients in the low Hb subgroup exhibited a steady increase in Hb levels. This increase in Hb was observed in all baseline treatment subgroups. Moreover, results remained consistent after taking into account age, race (Caucasian vs. other), disease duration, DAS28-CRP, seropositivity, and smoking status using multivariable regression analysis (data not shown).

**Fig. 2:**
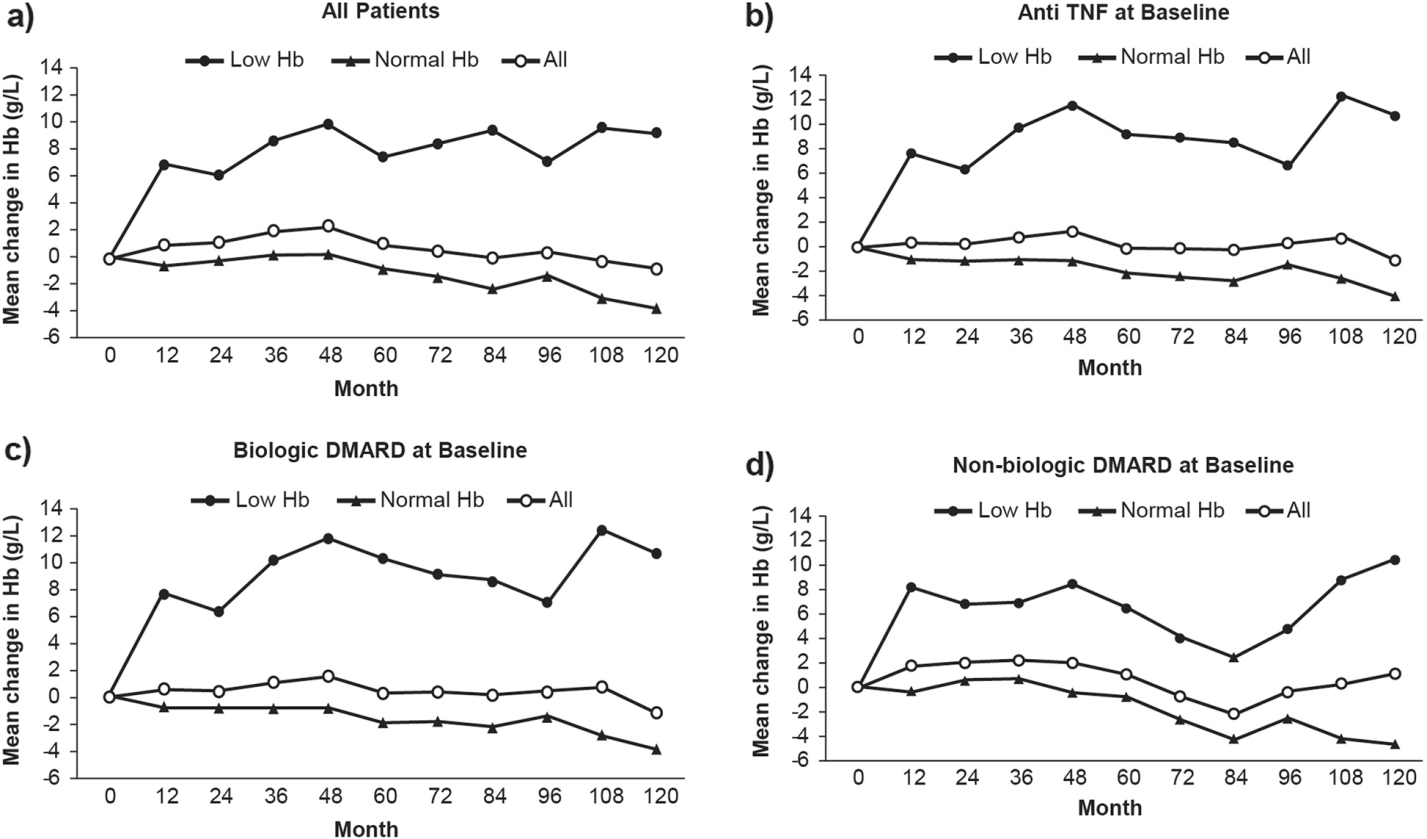
Mean change in Hb over time by Hb subgroup DMARD, disease-modifying anti-rheumatic drug; Hb, hemoglobin; TNF, tumor necrosis factor

At week 120, the mean Hb from baseline increased by 9.24 g/L in patients with low Hb (*n* = 42) at baseline, whereas it decreased by 3.74 g/L in patients with normal Hb (*n* = 144). Similarly, patients with low Hb taking an anti-TNF, biologic DMARD, or non-biologic DMARD at baseline also demonstrated an increase in Hb levels over 10 years (Fig. 2). However, patients with normal Hb showed no to minor changes in Hb levels throughout 10 years.

#### Hemoglobin over time by current medication at baseline subgroup

**Fig. 3:**
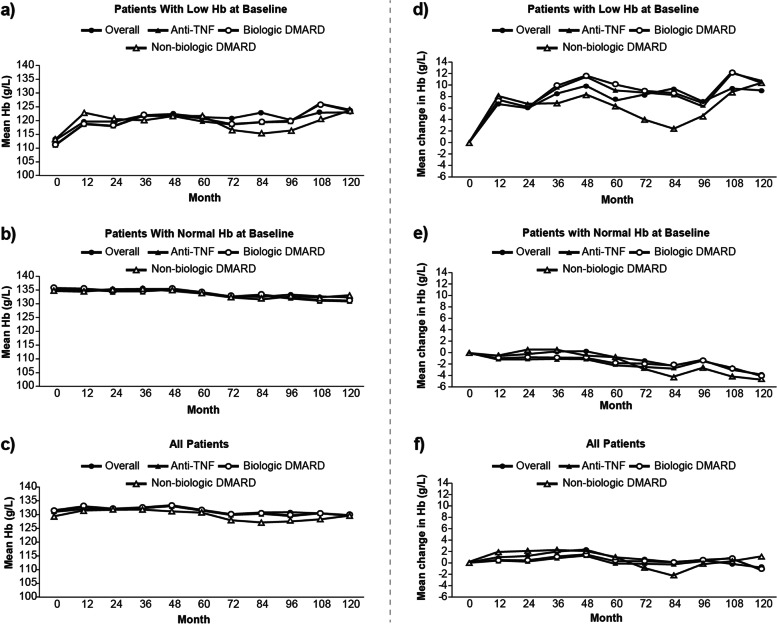
**a**–**c** Mean Hb and **d**–**f** mean change over time by current medication at baseline subgroup DMARD, disease-modifying anti-rheumatic drug; Hb, hemoglobin; TNF, tumor necrosis factor

Overall, patients with low Hb at baseline reported an increase in mean Hb levels from baseline to 10 years (Fig. 3a–c); a similar trend was noted regardless of class of current medication. Patients with normal Hb at baseline showed steady Hb levels throughout the 10-year study period.

Overall, an improvement in the mean Hb from baseline to month 120 was observed in patients taking non-biologic DMARDs (Fig. 3d–f).

### Total sharp score over time: hemoglobin subgroups and current medication at baseline

#### Total sharp score by hemoglobin subgroups

**Fig. 4:**
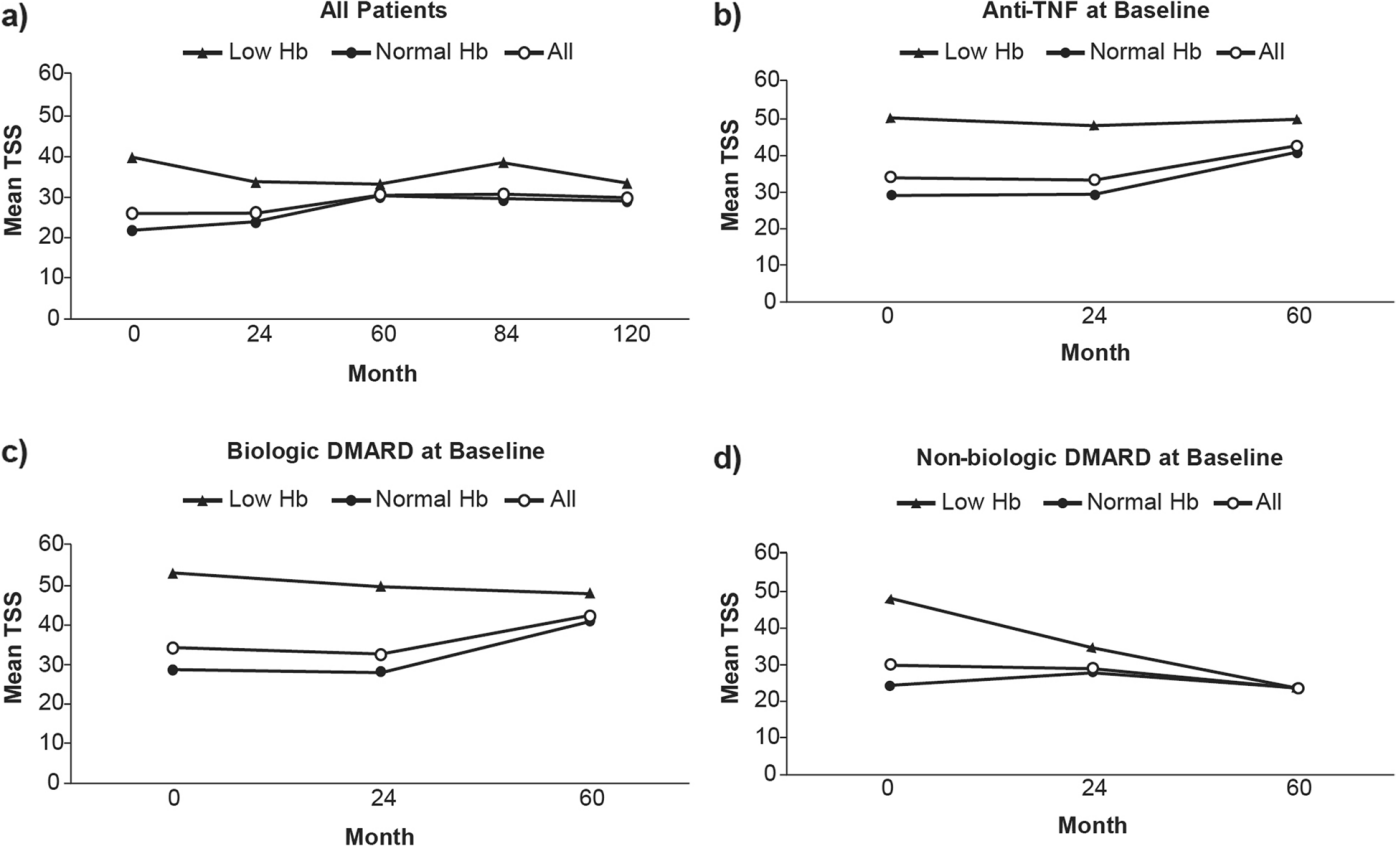
Mean TSS over time by Hb subgroup DMARD, disease-modifying anti-rheumatic drug; Hb, hemoglobin; TNF, tumor necrosis factor; TSS, total sharp score

**Fig. 5:**
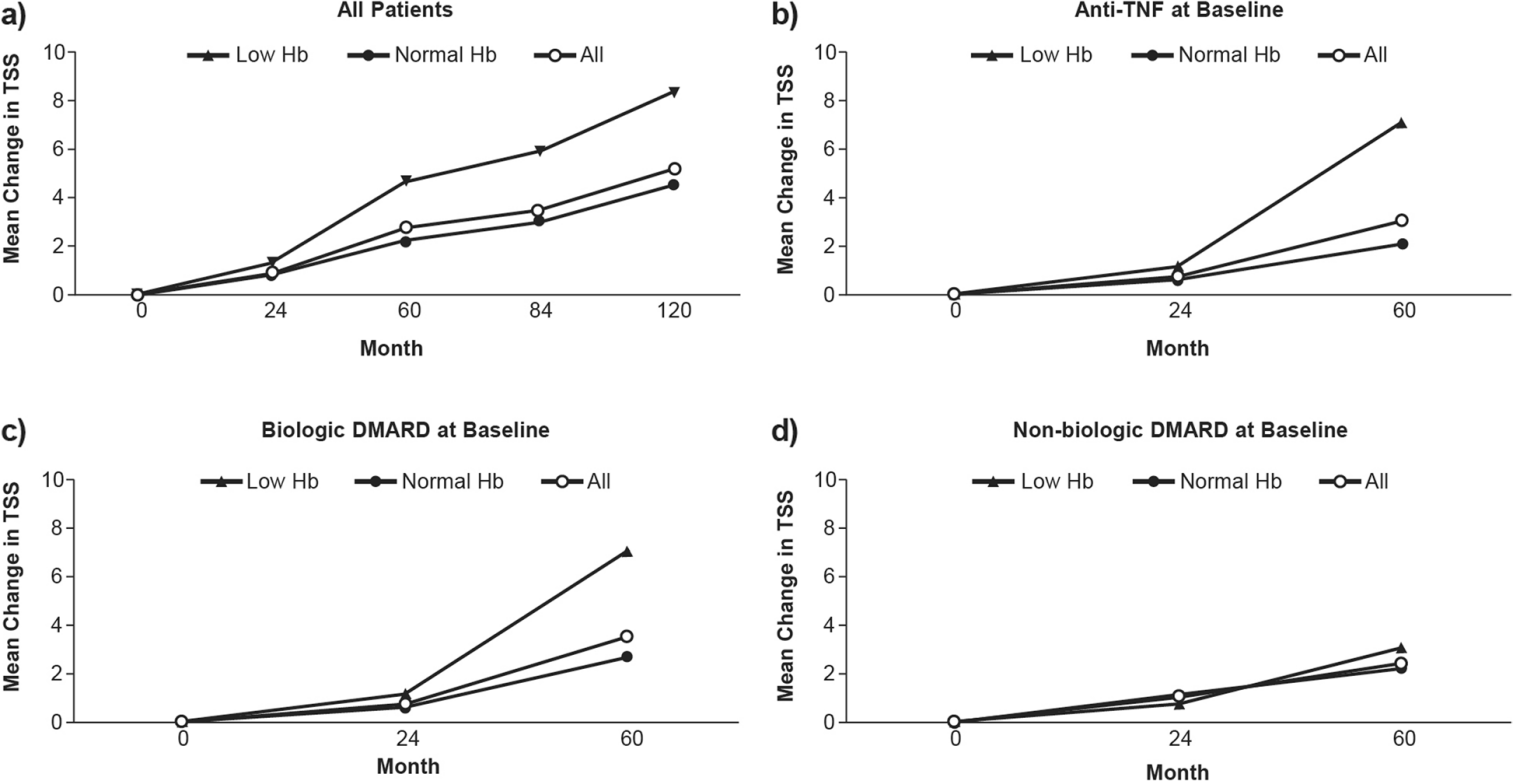
Mean change in TSS over time by Hb subgroup DMARD, disease-modifying anti-rheumatic drug; Hb, hemoglobin; TNF, tumor necrosis factor; TSS, total sharp score

Overall, the mean TSS at each time point was higher in patients with low Hb at baseline (40.05 vs 21.91) through month 120 (33.32 vs 29.01) compared with patients with normal Hb, respectively (Fig. 4); this was true for both absolute values and the mean change from baseline (Fig. 5). For over 60 months, no to minor improvements in TSS were observed for patients with low Hb taking either an anti-TNF or biologic DMARD; whereas a decrease was observed with non-biologic DMARD use (Fig. 4). A minor increase in TSS was observed for patients with normal Hb taking an anti-TNF or biologic DMARD from month 24 to month 60, whereas TSS was reduced for patients with low and normal Hb taking non-biologic DMARDs. Overall, mean changes in TSS from baseline to 10 years in patients with low Hb (*n* = 12; 8.38) were greater than for patients with normal Hb (*n* = 56; 4.53) (Fig. 5). At months 84 and 120, the anti-TNF and biologic DMARD low Hb cohorts consist of the same patients. Patients with low Hb at baseline and currently either on an anti-TNF or biologic DMARD showed no to minor changes in the mean TSS throughout 60 months; however, those on non-biologic DMARD demonstrated reduced TSS. Furthermore, no to minor differences were detected in the mean change in TSS over time by current medication at baseline in patients with normal Hb at baseline (Supplementary Figs. 1 and 2). Moreover, results remained consistent after taking into account age, race (Caucasian vs. other), disease duration, DAS28-CRP, seropositivity, and smoking status using multivariable regression analysis (data not shown).

## Discussion

The BRASS registry data reveal that low Hb could be an independent and important factor associated with radiographic progression/damage and may be used to monitor chronic conditions, such as RA. Our observations are in line with a previously published study, which showed that anemia is independently associated with radiographic progression in RA and may be involved in the subclinical inflammation of RA [5].

Throughout 10 years, patients with low Hb continued to have lower Hb and greater radiographic progression than patients with normal Hb. Post hoc analyses of the PREMIER trial data reported that MTX-treated RA patients with low Hb levels were significantly associated with faster radiographic progression/damage over a 2-year study period [9]; also the study suggested that low Hb was a valuable DAS28-CRP-independent indicator of joint damage progression. We also observed that patients with low Hb demonstrated a greater increase in the mean Hb levels over time compared with patients with normal Hb; the same was observed regardless of class of medication. Hashimoto et al. [11] reported that RA patients treated with tocilizumab showed a greater improvement in low Hb levels, whereas the change in the CDAI was a predictor of Hb change. A pooled analysis from three large randomized clinical trials reported a better response to anti-TNF (infliximab + MTX) in terms of normalization of Hb levels in RA patients with anemia at baseline [12]. Likewise, improvement in the iron-related parameters, such as mean Hb, serum iron, and mean corpuscular volume, in RA patients with anemia (mean Hb: 11.3 g/dL) has been reported after tocilizumab treatment [13, 14].

In the current study, patients with low Hb had a greater TSS than patients with normal Hb, indicating greater radiographic progression throughout 10 years. Similarly, a population-based longitudinal real-world study reported the link between anemia and disease activity, medications, and radiographic progression in comparison with non-anemic counterparts. This study demonstrated that radiographic progression rates were increasing with the severity of anemia, and this effect was maintained in subgroups of patients treated with anti-TNF or corticosteroids, without non-selective NSAIDs [5]. Furthermore, greater disease severity and risk of comorbidities were observed in patients with low Hb compared with patients with normal Hb enrolled in the registry (*n* = 10,397) [7].

A strength of this study is a patient population with longer disease duration (> 10 years) and receiving treatment of various classes of RA medications including biologic DMARDs and non-biologic DMARDs, which would make these results broadly applicable in the clinical practice. Furthermore, mean Hb levels and changes in Hb levels were assessed over a longer duration (10 years) compared with previously published studies.

The BRASS study has some limitations. First, the BRASS being a single-center registry with geographical limitations on patient availability and retention, the generalizability of the results may need further validation. Second, the study did not explore whether changes in Hb levels and TSS over time differ with continuous treatment of specific classes of medication. Third, we did not analyze surrogate measures (CRP or erythrocyte sedimentation rate) to correlate with the radiographic disease progression. Fourth, we did not determine the mechanisms behind the interaction of Hb under different treatment modalities. In addition, these analyses were retrospective in nature. Hence, further studies will be needed to comprehensively understand the effect of different classes of medications on RA patients with low Hb. Lastly, in the present study, we did not determine the overall iron status by assessing ferritin, hepcidin, and mean corpuscular volume.

## Conclusions

The real-world data from the BRASS registry demonstrate that patients with low Hb at baseline continued to have lower Hb throughout 10 years of follow-up. Sustained improvements in Hb were observed for patients with low Hb treated with any class of DMARDs. Patients with low Hb at baseline tend to have a greater rate of radiographic progression as measured by TSS compared with RA patients with normal Hb levels. Moreover, no meaningful TSS difference was observed in low Hb patients comparing treatment with conventional synthetic vs biologic DMARDs. Future analyses may evaluate constant therapy and non-constant therapy cohorts and disease severity cohorts.

## Supplementary Information

**Additional file 1: Supplementary Figure 1.** Mean TSS over time by current medication at baseline subgroup. **Supplementary Figure 2.** Mean change in TSS over time by current medication at baseline subgroup.

## Data Availability

All data generated or analyzed during this study are included in this published article [and its supplementary information files.

## Abbreviations

BRASS: Brigham and Women’s Rheumatoid Arthritis Sequential Study
CDAI: Clinical Disease Activity Index
CRP: C-reactive protein
DAS28: Disease Activity Score in 28 joints
Hb: Hemoglobin
MBDA: Multi-biomarker disease activity
MTX: Methotrexate
NSAID: Nonsteroidal anti-inflammatory drug
QMs: Quality measures
RA: Rheumatoid arthritis
SD: Standard deviation
TNF: Anti-tumor necrosis factor
TSS: Total Sharp score
WHO: World Health Organization
