# Supplementary material for: Association of hemoglobin levels with radiographic progression in patients with rheumatoid arthritis: an analysis from the BRASS registry
Source: Arthritis Res Ther. 2023 May 27;25:88. doi: 10.1186/s13075-023-03068-w (PMC10224608; doi:10.1186/s13075-023-03068-w)
Supplement: Supplementary file 1 — Additional file 1: Supplementary Figure 1. Mean TSS over time by current medication at baseline subgroup. Supplementary Figure 2. Mean change in TSS over time by current medication at baseline subgroup. [file 13075_2023_3068_MOESM1_ESM.docx]

**Association of hemoglobin levels with radiographic progression in patients with rheumatoid arthritis: an analysis from the BRASS Registry**

Nancy Shadick^1*^, Owen Hagino^2^, Amy Praestgaard^3^, Stefano Fiore^3^, Michael Weinblatt^1^, Gerd Burmester^4^

^1^Division of Rheumatology, Inflammation, and Immunity, Brigham and Women’s Hospital, Boston, MA, United States of America

^2^Sanofi, Research and Development, Bridgewater, NJ, United States of America

^3^Sanofi, Global Medical Affairs, Biostatistics, Cambridge, MA, United States of America

^4^Department of Rheumatology and Clinical Immunology, Charité – Universitätsmedizin, Berlin, Germany

**Corresponding author’s name, address, and email:**

Name**:** Nancy Shadick

Address: Division of Rheumatology, Inflammation, and Immunity, Brigham and Women’s Hospital, 75 Francis Street, Boston, MA, United States of America.

Phone: +1-(617) 732-5266;

Email: [nshadick@bwh.harvard.edu](mailto:nshadick@bwh.harvard.edu).

**Supplementary Figure 1: Mean TSS over time by current medication at baseline subgroup**


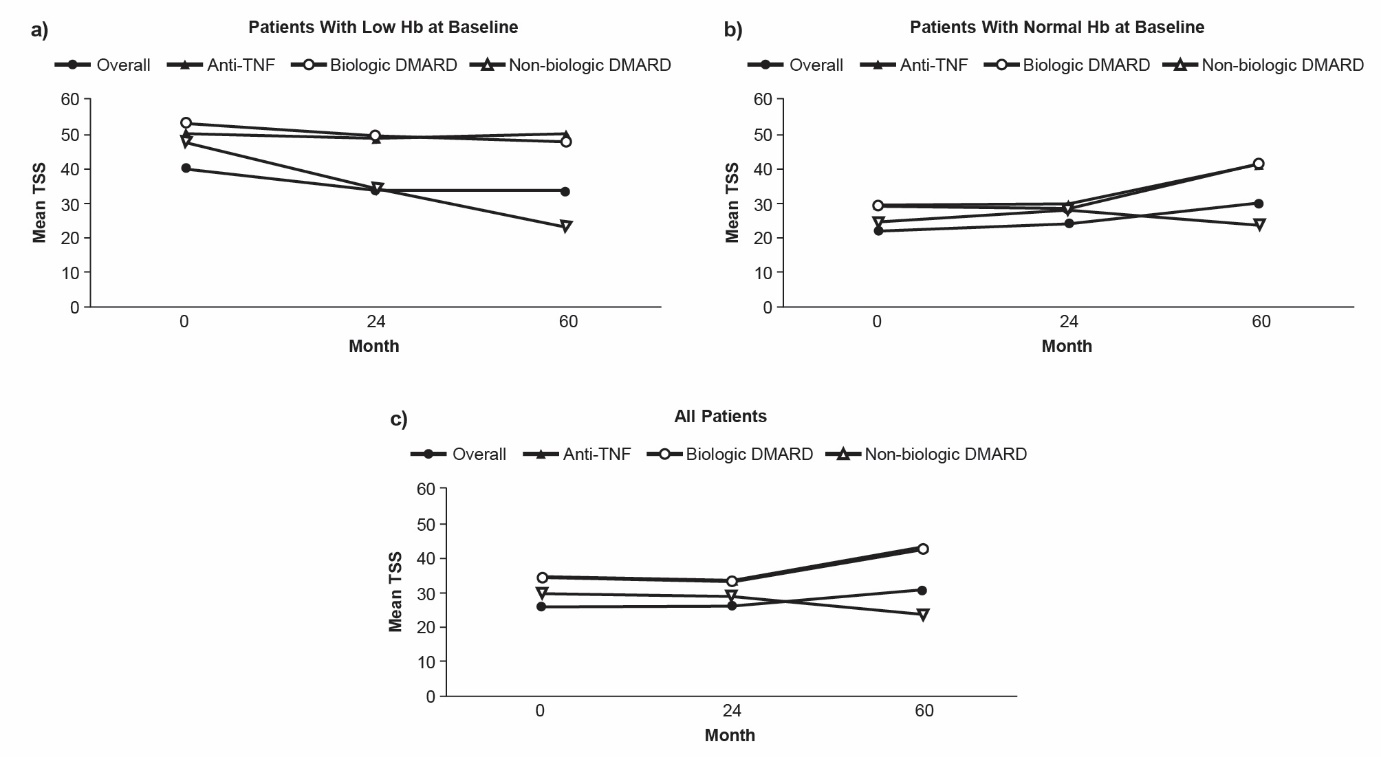
DMARD, disease-modifying anti-rheumatic drug; Hb, hemoglobin; TNF, tumor necrosis factor; TSS, total sharp score

**Supplementary Figure 2: Mean change in TSS over time by current medication at baseline subgroup**


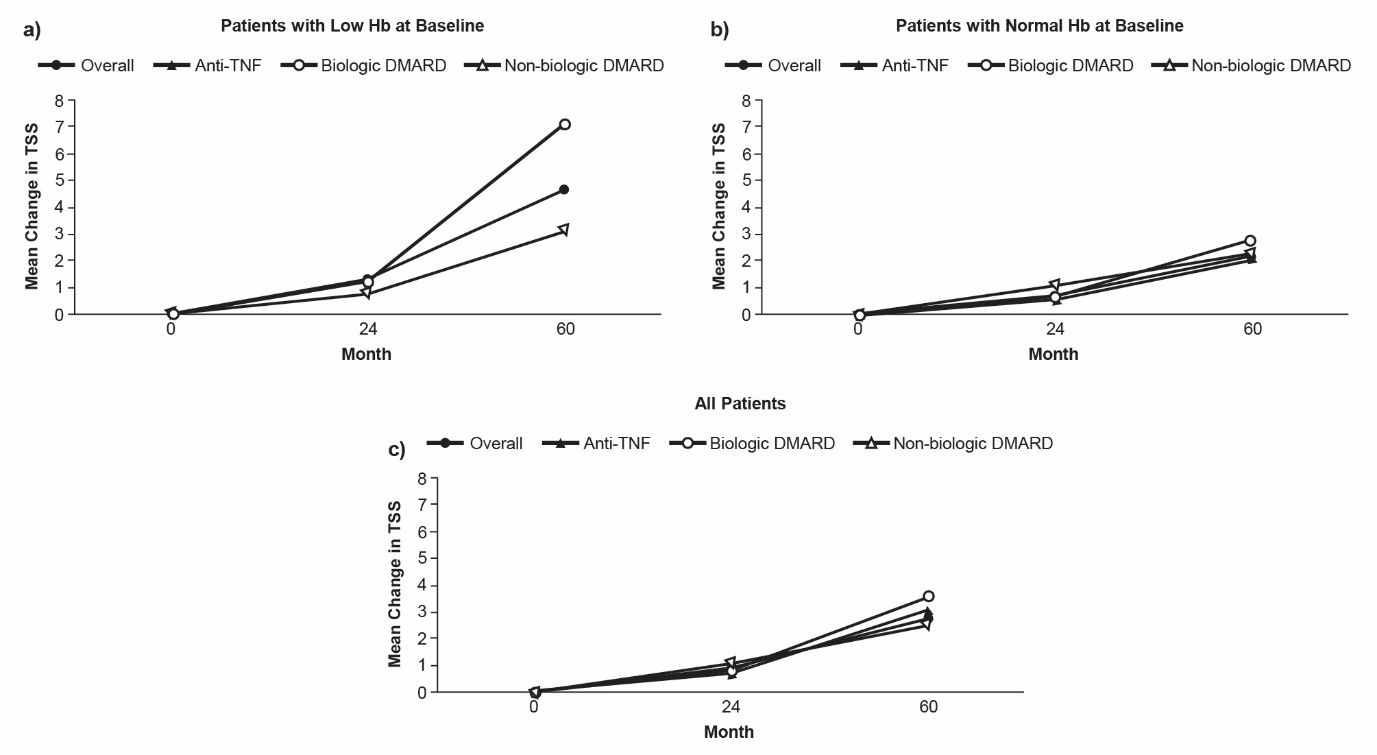
DMARD, disease-modifying anti-rheumatic drug; Hb, hemoglobin; TNF, tumor necrosis factor; TSS, total sharp score
